# Blood Oxidative Stress Marker Aberrations in Patients with Huntington's Disease: A Meta-Analysis Study

**DOI:** 10.1155/2020/9187195

**Published:** 2020-09-08

**Authors:** Quan Tang, Hua Liu, Xiao-Jie Shi, Yong Cheng

**Affiliations:** Key Laboratory of Ethnomedicine for Ministry of Education, Center on Translational Neuroscience, College of Life and Environmental Sciences, Minzu University of China, Beijing, China 100081

## Abstract

Huntington's disease (HD) is a hereditary autosomal dominant neurodegenerative disease. Although studies have shown that blood oxidative stress markers are dysregulated in HD patients, clinical data on the blood oxidative stress markers of HD patients is inconsistent. To better understand the pathogenesis of HD, we performed a systematic review and meta-analysis of blood oxidative stress markers in HD patients and healthy control (HC) subjects. A database search from PubMed and Web of Science identified 12 studies with 375 HD patients and 447 HC subjects in this meta-analysis. A random-effects meta-analysis showed that blood lipid peroxidation products (Hedges' *g* = 0.883, 95%CI = 0.637 to 1.130, *p* < 0.001), 8-hydroxyguanosine (Hedges' *g* = 1.727, 95%CI = 0.489 to 2.965, *p* = 0.006) levels, and the activity of glutathione peroxidase (Hedges' *g* = 2.026, 95%CI = 0.570 to 3.482, *p* = 0.006) were significantly increased in HD patients compared to controls. In contrast, reduced glutathione levels were lower in HD patients than in controls (Hedges' *g* = −0.611, 95%CI = −1.016 to − 0.207, *p* = 0.003). However, blood superoxide dismutase, cholesterol, high-density lipoproteins, low-density lipoproteins, and triglycerides did not show significant differences between cases and controls. Taken together, this study clarified the associations between blood oxidative stress markers and HD, supporting the clinical evidence that HD is accompanied by increased oxidative stress.

## 1. Introduction

Huntington's disease (HD), also known as Huntington's chorea, is a hereditary, autosomal, and dominant neurodegenerative disease; the disease is caused by a dominantly inherited cytosine-adenine-guanine (CAG) trinucleotide repeat expansion in the huntingtin gene, on chromosome IV [1, 2]. Patients with HD usually show a progressive decline of motor and cognitive functions and have a typical disease duration of 15–20 years [3, 4]. Additionally, HD is the most common monogenic neurological disorder in the developed world, with a prevalence of 10.6–13.7 individuals per 100,000 in Western populations [5]. Currently, there is no effective treatment for this devastating disease, and it is a burden to society, for affected individuals, and their families. Therefore, it is important to better understand the pathophysiology of HD, and subsequently develop an effective treatment for the disease.

Increasing evidence suggests that oxidative stress is a primary event in the neuropathology of neurodegenerative diseases [6, 7]. Oxidative stress can be defined as an imbalance between oxidants and antioxidants, in favor of oxidants, resulting in cell damage, dysfunction, or death. The main antioxidant enzymes are superoxide dismutase (SOD), catalase, and glutathione peroxidase (GPx). The primary final products of lipid peroxidation are malondialdehyde (MDA) and trans-4-hydroxy-2-nonenal (4-HNE), and 8-hydroxydeoxyguanosine (8-OHdG) is an important marker for DNA damage [8]. Previous studies have reported that blood oxidative stress markers were dysregulated in patients with neurodegenerative diseases, and that the related markers had the potential to inform the diagnosis of these diseases. However, the clinical data from these studies were largely inconsistent. To address the inconsistent data, meta-analyses have been performed; significant associations between Alzheimer's Disease (AD), Parkinson's Disease (PD), Amyotrophic Lateral Sclerosis (ALS), and blood oxidative stress markers were found, and these markers included MDA, 8-OHdG, SOD, and GPx [8–10]. Moreover, studies have suggested oxidative stress marker aberrations in patients with HD. However, there were inconsistent clinical data in the blood oxidative stress markers of HD. For example, Ciancarelli et al. found that blood SOD activity was higher in HD patients than in controls [11], and the data from Klepac et al. did not show a significant difference between patients with HD and healthy control (HC) subjects in terms of SOD activity [12]. Tunez et al. even suggested that the activity of SOD was significantly decreased in HD patients compared to HC subjects [13]. Due to the inconsistent data, a meta-analysis on the association between blood oxidative stress markers and HD is necessary.

In this study, to better understand the pathogenesis of HD, we conducted a meta-analysis of the measurements of oxidative stress products and antioxidants in the peripheral blood of patients with HD as well as in HC subjects.

## 2. Materials and Methods

We followed the methods of Chen et al. 2018 [14], since the two papers used a similar research strategy and the same statistical analysis methods.

### 2.1. Search Strategy and Study Selection

We manually performed a systematic search for studies of blood oxidative stress parameters in HD with PubMed, Web of Science, and China National Knowledge Infrastructure CNKI, up to January 2020. The search strategy was to search for “Huntington's disease” along with one of the following terms: “oxidative stress,” “superoxide dismutase,” “malondialdehyde,” “trans-4-hydroxy-2-nonenal,” “lipid peroxidation products,” “glutathione,” “catalase,” “glutathione peroxidase,” “8-OHdG,” “cholesterol”, “HDL”, “LDL,” or “triglycerides.” Peer-reviewed English or Chinese articles that reported data on the concentrations of oxidative stress markers in HD patients and HC subjects were included. Exclusion criteria were as follows: (1) no necessary data; (2) oxidative stress markers were measured in animal models; (3) no HC subjects; (4) samples overlapped with other studies; (5) *in vitro* data; (6) patients suffering from serious complications; (7) samples were not from blood; and (8) individual markers were studied in less than three articles.

### 2.2. Data Extraction

One investigator (QT) extracted the data, which were independently verified by another investigator (HL). Data on blood oxidative stress marker concentrations or activities, *p* values, sample sizes, and standard deviations for cases and controls were extracted to generate effective sizes (ESs). Data on age, sex, CAG repeat number (CAG-RN), country, publication year, sampling source, and diagnosis were also extracted (Table 1).

### 2.3. Statistical Analysis

We used the Comprehensive Meta-analysis Version 2 software to perform all the statistical analyses. ESs were mainly generated using sample sizes, mean concentrations, and standard deviations (SD), or sample sizes and *p* values if the data for the mean concentrations and SD were not available. The standardized mean differences in oxidative stress marker concentration between HD patients and HC subjects were calculated as ESs, which can be converted to Hedge's *g*, which provides unbiased ESs adjusted for sample sizes [15]. We calculated an ES estimate for each oxidative stress biomarker assessed in the studies included in the meta-analysis. A random-effects model was chosen for the meta-analysis because it is a more conservative model, meaning there is significance between-study heterogeneity [16].

The between-study heterogeneity was assessed using the Cochrane *Q* test and *I*^2^ statistic [17]. A *p* value < 0.10 was considered statistically significant for the Cochrane *Q* test. The inconsistency across studies was decided by the *I*^2^ index in order to evaluate the impact of the heterogeneity; 0.25 < *I*^2^ < 0.5 indicated low levels of between-study heterogeneity; 0.5 ≤ *I*^2^ < 0.75 indicated moderate levels of between-study heterogeneity; and *I*^2^ ≥ 0.75 indicate high levels of between-study heterogeneity. In addition, potential publication bias was determined by Egger's test, which assessed the asymmetry of the funnel plot.

Statistical significance of this meta-analysis was set at *p* < 0.05, except where noted.

## 3. Results

**Figure 1:**
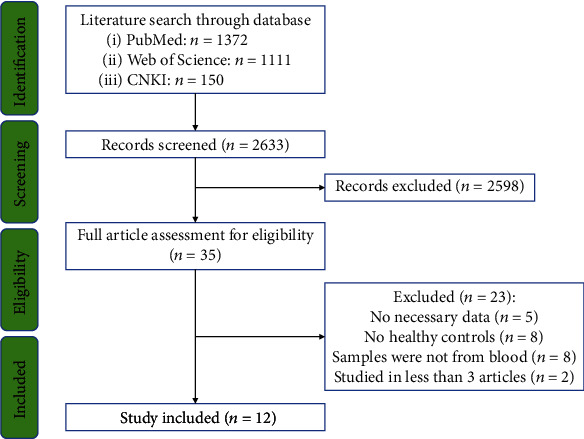
PRISMA flowchart of the literature search.

Firstly, the systematic search through the databases yielded 1,372, 1,111, and 150 records form PubMed, Web of Science, and CNKI, respectively. After a preliminary screening based on titles and abstracts, 35 articles relevant to this study were selected for full text scrutiny. After scrutiny, 23 studies were excluded due to the following reasons: no necessary data (*n* = 5); no HC subjects (*n* = 8); samples were not from blood (*n* = 8); and individual markers were studied in less than three articles (*n* = 2). In the end, 12 eligible articles [11–13, 18–26] were included in the meta-analysis, as shown in Figure 1.

### 3.1. Association of HD with Blood Oxidative Stress Markers

**Figure 2:**
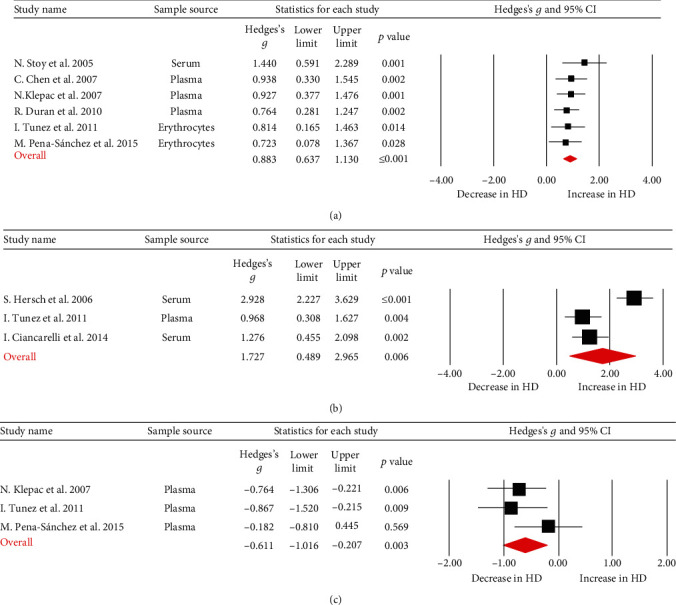
Studies of blood LPO, 8-OHdG, and GSH in Huntington's disease. Forest plot displaying random-effects meta-analysis results of the association between LPO (a), 8-OHdG (b), GSH (c), and Huntington's disease. The sizes of the squares are proportional to study weights.

Lipid peroxidation product (LPO) was reported in six articles, and all six studies showed higher LPO levels in HD patients than in HC subjects. Moreover, the sampling source of one study was serum, three were plasma, and two were erythrocytes. We conducted a meta-analysis of these six studies and observed a significant increase in LPO levels in the HD patients compared to their respective controls (Figure 2(a); Hedges' *g* = 0.883, 95%CI = 0.637 to 1.130, *p* < 0.001). Next, we performed subgroup analyses, and the results showed that LPO levels were significantly increased in both the erythrocytes (Figure S1; Hedges' *g* = 0.768, 95%CI = 0.311 to 1.226, *p* = 0.001) and plasma (serum) (Figure S1; Hedges' *g* = 0.930, 95%C I = 0.638 to 1.223, *p* < 0.001) from HD patients compared to their respective controls.

Moreover, three papers that reported 8-OHdG levels showed higher levels of 8-OHdG in patients with HD. The sample sources were plasma or serum. The meta-analysis showed that the levels of 8-OHdG in HD patients were significantly higher than in HC subjects (Figure 2(b); Hedges' *g* = 1.727, 95%CI = 0.489 to 2.965, *p* = 0.006).

Reduced glutathione (GSH) levels were also measured in three papers, and all samples were from plasma. The meta-analysis demonstrated that GSH levels were significantly lower in patients with HD than in controls (Figure 2(c); Hedges' *g* = −0.611, 95%CI = −1.016 to − 0.207, *p* = 0.003).

**Figure 3:**
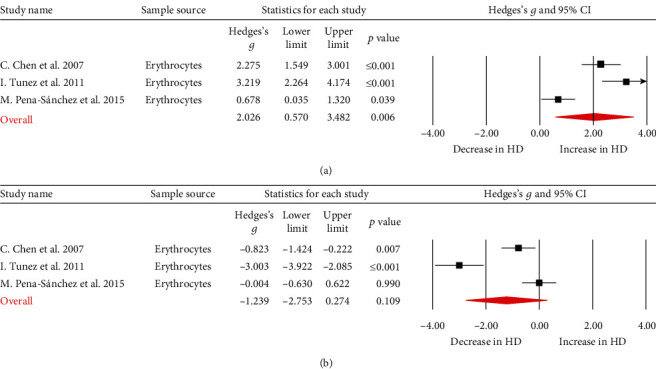
Studies of erythrocyte GPx and SOD in Huntington's disease. Forest plot displaying random-effects meta-analysis results of the association between GPx (a), SOD (b), and Huntington's disease. The sizes of the squares are proportional to study weights.

Additionally, three papers reported GPx activity in HD patients, and the sampling source was erythrocytes. In contrast to the findings related to GSH, the meta-analysis found that GPx activity was significantly higher in patients with HD than in controls (Figure 3(a); Hedges' *g* = 2.026, 95%CI = 0.570 to 3.482, *p* = 0.006).

A total of five articles reported SOD activity; three of the sampling sources were from erythrocytes, one was from plasma, and one was from serum. We performed a meta-analysis on the three studies which analyzed erythrocyte SOD activity, and the results showed that there was no significant difference in SOD activity between the patients and the controls (Figure 3(b); Hedges' *g* = −1.239, 95%CI = −2.753 to 0.274, *p* = 0.109).

**Figure 4:**
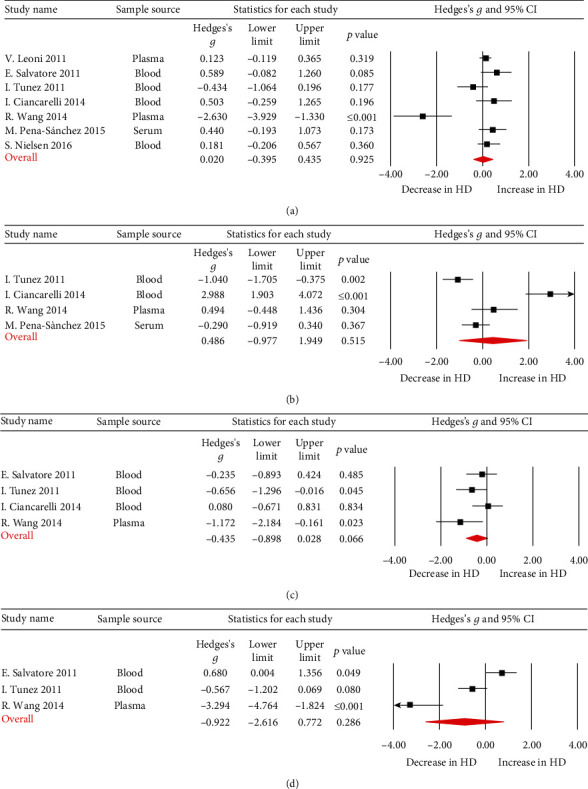
Studies of blood cholesterol, HDL, LDL, and triglycerides in Huntington's disease. Forest plot displaying random-effects meta-analysis results of the association between cholesterol (a), triglycerides (b), HDL (c), LDL (d), and Huntington's disease. The sizes of the squares are proportional to study weights.

The meta-analysis of other four oxidative stress-related factors, cholesterol (Figure 4(a); Hedges' *g* = 0.020, 95%CI = −0.395 to 0.435, *p* = 0.925), triglycerides (Figure 4(b); Hedges' *g* = 0.486, 95%CI = −0.977 to 1.949, *p* = 0.515), HDL (Figure 4(c); Hedges' *g* = −0.435, 95%CI = −0.898 to 0.028, *p* = 0.066), and LDL (Figure 4(d); Hedges' *g* = −0.922, 95%CI = −2.616 to 0.772, *p* = 0.772), did not show significant differences between HD patients and HC subjects.

### 3.2. Investigation of Heterogeneity

Through *Q* test and *I*^2^ test for the nine blood oxidative stress markers, it was found that LPO did not show between-study heterogeneity, while GSH and HDL showed low levels of between-study heterogeneity. However, 8-OHdG, SOD, GPx, LDL, cholesterol, and triglycerides showed high levels of between-study heterogeneity. Furthermore, the results of Egger's test showed that all markers in the meta-analysis had no significant publication biases (Table 2).

## 4. Discussion

To the best of our knowledge, this meta-analysis is the first work to pool data from studies evaluating blood oxidative stress marker levels in HD patients and compare these to controls. We summarized 12 studies with 375 HD patients and 447 HC subjects and measured nine blood oxidative stress markers including LPO, 8-OHdG, GSH, GPx, SOD, cholesterol, HDL, LDL, and triglycerides. There were significant increases in the concentrations or activities of LPO, 8-OHdG, and GPx of patients with HD. In contrast, GSH levels were significantly decreased in HD patients. The levels of the other oxidative stress markers did not show significant differences between the HD patients and controls. In addition to the aberrations in oxidative stress marker levels in HD, studies have reported oxidative stress marker levels in presymptomatic gene carriers (pre-HD). Klepac et al. found that pre-HD patients had higher plasma LPO and lower plasma GSH levels than HC subjects, whereas LPO and GSH levels did not show significant differences between pre-HD and patients with HD [22], suggesting that oxidative stress occurs before the onset of the HD symptoms. In contrast, data from Duran et al. showed that LPO concentrations were significantly elevated in patients with HD, but not in pre-HD [20]. Furthermore, evidence suggests that oxidative stress marker levels may associate with the disease severity of HD. One study demonstrated that plasma MDA levels were significantly correlated with the disease severity of HD, and MDA levels were higher in HD patients with a moderate disease severity than in patients with a mild disease severity [18]. Another study revealed that oxidative stress marker levels were increased in patients with HD when compared with controls, and the effects were more intense in HD patients with a moderate disease severity than in patients with a mild disease severity [13]. Taken together, these results suggest a critical role of oxidative stress in the pathogenesis of HD and highlight the fact that continued investigations into the functional involvement of oxidative stress in HD onset and/or development are necessary.

LPO levels are based on the detection of MDA and 4-HNE, which have strong mutagenicity and cytotoxicity [27]. In animal experiments, Verma et al. demonstrated that the reduction of lipid peroxidation is an important reason to relieve abnormal behavior in the QA-induced HD model [28], and data from Lee et al. showed that the modulation of lipid peroxidation and mitochondrial function improves neuropathology in HD mice [29]. Additionally, Skouta et al. suggested that lipid peroxidation mediates Huntington's disease phenotypes and found that the drug inhibited oxidative lipid peroxidation and reduced cell death in cellular models of Huntington's disease (HD) [30]. Although how lipid peroxidation contributes to the onset and/or development of HD is unclear, it was suggested that lipid peroxidation induced by DNA damage might play an important role. In fact, mounting evidence has demonstrated that the complex family of LPO products gives rise to a variety of DNA adducts and cause DNA damage [7, 31, 32]. In addition, mutant huntingtin provokes oxidative damage to nuclear and mitochondrial DNA [33]. Acevedo-Torres et al. demonstrated that mitochondrial DNA damage is a hallmark of chemically induced and the R6/2 transgenic model of HD [34]. Moreover, Browne et al. found that increased oxidative damage to nuclear DNA, in the form of 8-OHdG, occurs in the postmortem caudate in HD patients [35]. In this study, the outcome of the meta-analysis indicated significantly higher levels of lipid damage markers (LPO) and DNA damage markers (8-OHdG) in the patients with HD as compared to controls. Therefore, our clinical data together with preclinical results indicated that lipid peroxidation in a DNA damage pathway may play a critical role in the pathogenesis of HD.

It is well known that GSH is an important endogenous antioxidant, and studies have implicated GSH redox imbalances in neurological diseases [36]. Our results showed that GPx activity was increased in patients with HD when compared with controls. In contrast, GSH levels were decreased in HD patients. One possible explanation for the differential changes in GPx and GSH in patients with HD is the different sample sources, given that the GPx data were from erythrocytes whereas the GSH data were from plasma. GPx are known to catalyze the reduction of H_2_O_2_ or organic hydroperoxides to water or the corresponding alcohols, respectively, typically using GSH as reductant [27], and as a result, two GSH molecules are oxidized into oxidized glutathione [37]. Therefore, it is likely that increased oxidative stress in HD patients causes decreased GSH levels, and the increased GPx activity was a compensatory mechanism to maintain homeostasis in the body.

In addition to the dysregulation of blood oxidative stress markers in patients with HD found in this study, other meta-analyses also showed significant associations between AD, PD, ALS, and blood oxidative stress markers. Similar to HD, blood levels of MDA and 8-OHdG were elevated in patients with AD, PD, and ASL [7–10, 38]. In contrast, in AD patients, GPx activity tended to decrease in plasma or serum [9]; in PD patients, GPx activity showed no significant differences, and GSH was significantly decreased when compared with controls [8]. Furthermore, GPx activity did not show significant difference between patients with ALS and controls, whereas decreased GSH levels were found in the peripheral blood of ALS patients [10]. Functionally, it has been proposed that vitamin B1 deficiency-mediated neurodegenerations (such as AD, PD, and HD) were at least partly due to the interplay between oxidative stress, ER stress, and autophagy [39]. This evidence implied that different neurodegenerative diseases have some shared and distinct oxidative stress responses.

The levels of heterogeneity ranged from small to high for the individual oxidative stress markers in the meta-analysis. The strength of this study is the small heterogeneity seen for LPO and GSH, suggesting robustness in the outcomes of the meta-analysis. However, high levels of between-study heterogeneities were found for the studies that analyzed 8-OHdG levels and GPx activity. The limitation of this meta-analysis is the relatively small number of studies evaluating the associations between 8-OHdG, GPx, and HD, therefore preventing us from analyzing potential confounders that contribute to the between-study heterogeneities. Another limitation of this study is that it may be difficult to observe significant differences between cases and controls for several oxidative stress markers due to the limited number of studies. In fact, there was a trend that HDL levels were decreased in patients with HD (Hedges' *g* = −0.435, 95%CI = −0.898 to 0.028, *p* = 0.066). Therefore, it is likely with increased sample size, the HDL levels would show significant differences between patients with HD and controls, and future studies are necessary to validate this hypothesis. The third limitation is that we focused on only blood oxidative stress markers; the levels of inflammation markers such as C-reactive protein and interleukin-6 have also been reported to be abnormal in patients with HD [40, 41], and the clinical data were inconsistent, which merits further exploration.

## 5. Conclusion

The findings of our meta-analysis demonstrated elevated peripheral blood concentrations or activity of LPO, 8-OhdG, and GPx and low GSH levels in HD patients. Additionally, there were no significant differences between HD patients and controls in terms of SOD, cholesterol, LDL, HDL, and triglyceride levels. This finding strengthens the clinical evidence that HD is accompanied by an abnormal oxidative stress response and clarifies the profile of the oxidative stress markers in patients with HD.

## Figures and Tables

**Table 1:** Characteristics of included studies measuring peripheral blood markers.

No.	Study and year	Country or region	Sample size	Gender (M/F)	Mean age (years)	CAG-RN	OS markers measured	Sample source	Diagnosis criteria
1	Stoy 2005	UK	HD 11	3/8	NA	NA	MDA and 4-hydroxynonenal; tryptophan metabolites	Serum	NA
			HC 15	4/11	NA	—			
2	Hersch 2006	USA	HD 32	NA	NA	NA	8-OHdG	Serum	UHDRS
			HC 32						
3	Chen 2007	Taiwan	HD 16	9/7	48.4	45.0	MDA; 8-OHdG; SOD; and GPx	Plasma, serum, and erythrocyte	UHDRS
			HC 36	19/17	53.0	—			
4	Klepac 2007	Croatia	HD 19	14/5	46	45.0	MDA; GSH; and SOD	Plasma	UHDRS
			HC 47	31/16	41	—			
5	Duran 2010	Spain	HD 24	10/14	48.4	45.1	LPO	Plasma	UHDRS
			HC 60	30/30	48.0	—			
6	Leoni 2011	Italy	HD 127	57/70	52.1	44.6	Cholesterol	Plasma	UHDRS
			HC 134	67/68	48.2	—			
7	Salvatore 2011	Italy	HD 17	10/7	48.8	44.8	Cholesterol; LDL; and HDL	Blood	UHDRS
			HC 17	10/7	49.2	—			
8	Tunez 2011	Spain	HD 19	9/10	43.5	46.5	8-OHdG; LPO; GSH; SOD; GPx; cholesterol; HDL; LDL; and triglycerides	Plasma, erythrocyte	UHDRS
			HC 19	8/11	42.2	—			
9	Ciancarelli 2014	Italy	HD 18	13/5	51.1	NA	8-OHdG; SOD; cholesterol; HDL; and triglycerides	Serum, plasma	UHDRS
			HC 10	4/6	50.0	—			
10	Wang 2014	USA	HD 8	5/3	57.6	42.5	Cholesterols; HDL; LDL; and triacylglycerols	Plasma	NA
			HC 8	5/3	57.0	—			
11	Peña-Sánchez 2015	Cuba	HD 14	8/6	50.0	45.0	Cholesterol; triglycerides; MDA; GSH; SOD; and GPx	Serum	UHDRS
			HC 29	12/17	48.0	—			
12	Nielsen 2016	Denmark	HD 70	36/33	51.5	43.3	Cholesterol	Blood	UHDRS
			HC 40	18/22	41.4	—			

HD: Huntington's disease; HC: healthy controls; M/F: male/female; CAG-RN: cytosine-adenine-guanine repeat number; OS: oxidative stress; MDA: malondialdehyde; LPO: lipid peroxidation products; 8-OHdG: 8-hydroxyguanosine; GSH: glutathione; GPx: glutathione peroxidase; SOD: superoxide dismutase; LDL: low-density lipoprotein; HDL: high-density lipoprotein; UHDRS: Unified Huntington's Disease Rating Scales; NA: not available.

**Table 2:** Summary of comparative outcomes for measurements of blood marker levels.

Marker	No. of studies	No. with HD/HC	Main effect		Heterogeneity				Publication bias	
			Hedges g (95% CI)	*p* value	*Q* statistic	df	*p* value	*I* ^2^ statistic	Egger's intercept	*p* value
LPO	6	103/206	0.883 (0.637–1.130)	≤0.001	2.221	5	0.818	0	2.65081	0.10766
8-OHdG	3	52/65	1.727 (0.489–2.965)	0.006	17.520	2	<0.001	88.585	-0.65530	0.98446
GSH	3	52/95	-0.611 (-1.016–-0.207)	0.003	2.690	2	0.261	25.639	2.80380	0.84614
GPx	3	49/84	2.026 (0.570–3.482)	0.006	21.812	2	<0.001	90.831	15.55092	0.27934
SOD	3	49/84	-1.239 (-2.753–0.274)	0.109	28.130	2	<0.001	92.890	-15.59612	0.29130
Cholesterol	7	273/257	0.020 (-0.395–0.435)	0.925	24.093	6	0.001	75.097	-1.37103	0.44288
HDL	4	62/54	-0.435 (-0.898–0.028)	0.066	4.656	3	0.199	35.565	-2.99853	0.54086
LDL	3	44/44	-0.922 (-2.616–0.772)	0.286	24.681	2	<0.001	91.897	-7.49790	0.40826
Triglycerides	4	59/66	0.486 (-0.977–1.949)	0.515	40.437	3	<0.001	92.581	12.84671	0.11963

df: degrees of freedom; HD: Huntington's disease; LPO: lipid peroxidation products; 8-OHdG: 8-hydroxyguanosine; GSH: glutathione; GPx: glutathione peroxidase; SOD: superoxide dismutase; LDL: low-density lipoprotein; HDL: high-density lipoprotein.

## Data Availability

The data used to support the findings of this study are included within the article.

## Supplementary Materials

Supplementary Materials Figure S1: subgroup analyses on LPO levels stratified by sampling source.
