# Supplementary material for: Blood Oxidative Stress Marker Aberrations in Patients with Huntington's Disease: A Meta-Analysis Study
Source: Oxid Med Cell Longev. 2020 Sep 8;2020:9187195. doi: 10.1155/2020/9187195 (PMC7499314; doi:10.1155/2020/9187195)
Supplement: Supplementary Materials — Figure S1: subgroup analyses on LPO levels stratified by sampling source. [file 9187195.f1.pptx]

## Slide 1
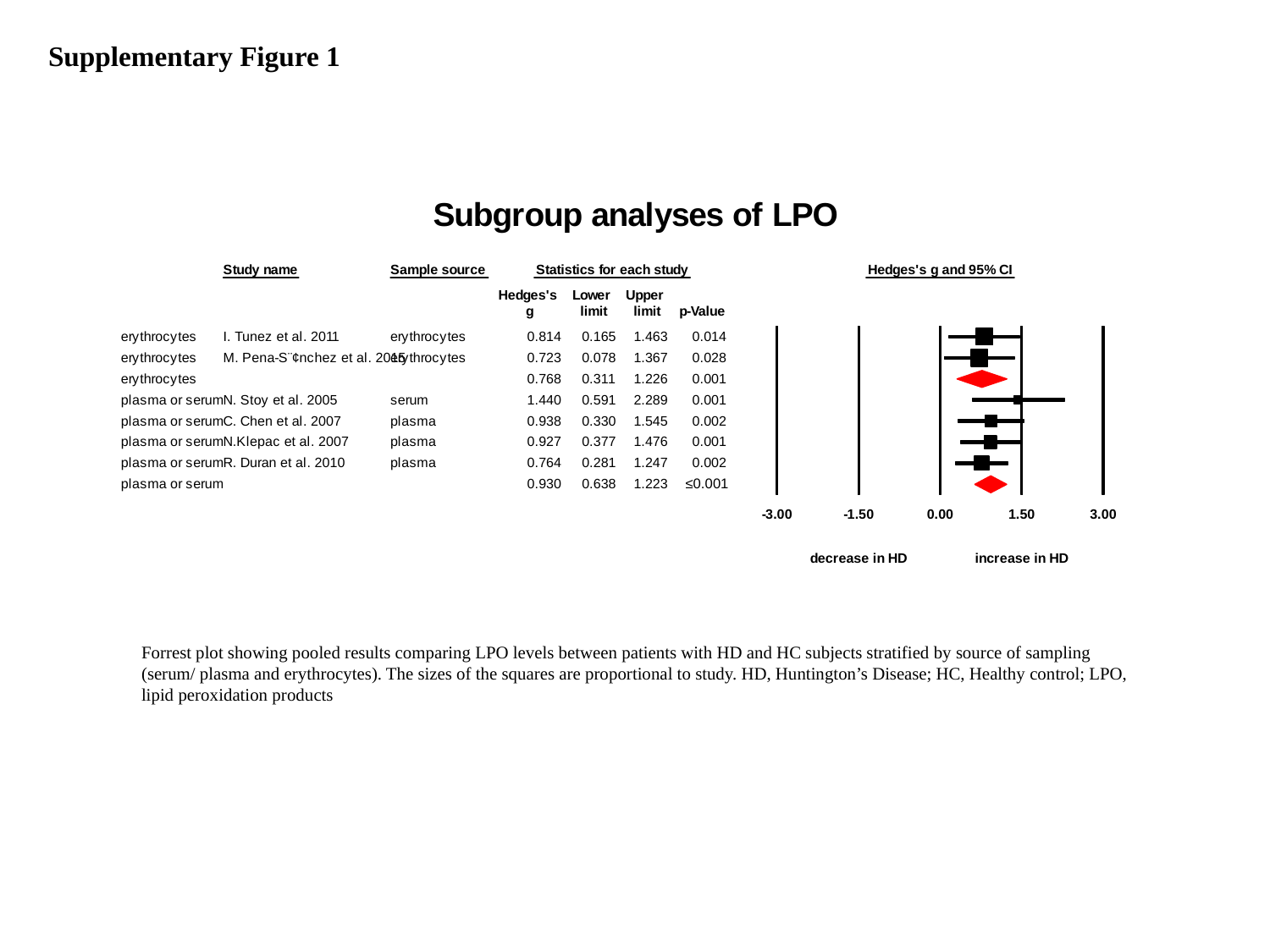

Supplementary Figure 1
≤0.001
Forrest plot showing pooled results comparing LPO levels between patients with HD and HC subjects stratified by source of sampling (serum/ plasma and erythrocytes). The sizes of the squares are proportional to study. HD, Huntington’s Disease; HC, Healthy control; LPO, lipid peroxidation products
